# Inhibitor Ranking through QM Based Chelation Calculations for Virtual Screening of HIV-1 RNase H Inhibition

**DOI:** 10.1371/journal.pone.0098659

**Published:** 2014-06-04

**Authors:** Vasanthanathan Poongavanam, Casper Steinmann, Jacob Kongsted

**Affiliations:** Department of Physics, Chemistry and Pharmacy, University of Southern Denmark, Odense M, Denmark; University of Pittsburgh, United States of America

## Abstract

Quantum mechanical (QM) calculations have been used to predict the binding affinity of a set of ligands towards HIV-1 RT associated RNase H (RNH). The QM based chelation calculations show improved binding affinity prediction for the inhibitors compared to using an empirical scoring function. Furthermore, full protein fragment molecular orbital (FMO) calculations were conducted and subsequently analysed for individual residue stabilization/destabilization energy contributions to the overall binding affinity in order to better understand the true and false predictions. After a successful assessment of the methods based on the use of a training set of molecules, QM based chelation calculations were used as filter in virtual screening of compounds in the ZINC database. By this, we find, compared to regular docking, QM based chelation calculations to significantly reduce the large number of false positives. Thus, the computational models tested in this study could be useful as high throughput filters for searching HIV-1 RNase H active-site molecules in the virtual screening process.

## Introduction

Predicting binding affinities as well as ranking several ligands with respect to each other is still a major challenge in computer-aided drug design, in particular in lead identification/optimization processes [1], [2]. For this, various biophysical methods have been used to accurately measure the binding affinity of various protein-ligand complexes [3]. However, these methods are generally too time consuming, expensive or inefficient to handle a large number of compounds. On the other hand, computational methods offer prediction of binding affinities at various levels of sophistication. These includes for example highly accurate ab initio free energy calculations (methods in this class are accuarate and computationally expensive) [4] or docking-based high efficient scoring functions (methods in this class are less accuarate but computationally inexpensive) such as force field (D-score) or empirical (Glide Score) scoring function as highlighted in a recent review [5]. From a virtual screening point of view, it is highly relevant to develop an affinity prediction method which is capable of both fast and relatively accurate screening of a large number of compounds [6]. The majority of the current scoring functions have been designed for virtual screening purposes. This means the aim is to distinguish binders from non-binders and not ranking of actives [5], [7]–[9].

Many drugs or inhibitors potentially bind with metal ions in the catalytic site of enzymes or receptors in order to exhibit their therapeutic effect, e.g., enzymes containing magnesium ions such as HIV-1 integrase and RNase H. Thus, a good scoring function needs to be able to accurately predict the metal-inhibitor interaction which impacts the overall binding affinity of the compounds. Although such metal-binding term is included in the scoring function e.g., in the Glide Score [8], the metal term considers only the anionic or highly polar interactions, therefore, ranking of actives might not appropriately be achieved [10].

It has previously been reported that magnesium ions in the HIV-1 reverse transcriptase associated ribonuclease H (RNase H or RNH) play an essential role in the binding and positioning of the RNA:DNA duplex (natural substrate) during digestion in the viral genome reverse transcription process [11], [12]. Inhibition of this enzyme by chelation of magnesium ions (active site binder) is indeed considered as an attractive drug target for AIDS therapy [11], [13]–[16].

Due to the importance of this chelation term in the overall binding affinity, we have here attempted to improve the binding affinity prediction through the use of quantum mechanical (QM) based calculation by primarily considering the chelation mechanism of inhibitors with the catalytically active magnesium ions. This could be useful as a high-throughput filter in virtual screening processes. Considering this chelation mechanism, two kinds of questions can be addressed using QM guided docking experiments: (1) can we improve the ranking of individual compounds based on the use of a scoring function? (2) can we improve the classification of binders and non-binder based on the scoring function using the chelation calculation? In order to address the above questions, we have tested docking simulations together with QM calculations based on both Møller–Plesset perturbation therory (MP2) and density functional theory (DFT) on a relatively large dataset. This dataset was retrieved from the literature and the PubChem database. In addition to addressing the above questions, we also used the QM based chelation calculation in the virtual screening process in order to validate the method. These calculations could be useful in order to reduce the number of false positives (i.e. inactive compounds being predicted as active compounds by the computational model).

## Computational Materials and Methods

### Binding free energy calculation

The binding of a ligand to a protein can be described by equation 1 below and the corresponding change in free energy/the binding energy (ΔG_Bind_) can thus be calculated as the difference between the free energies of the complex and ligand/protein (Eq. 2) all in aqueous solution.



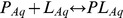
(Eq. 1)




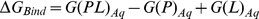
(Eq. 2)


Many scoring functions based on statistical or empirical methods are today used to approximate this binding energy, e.g., empirical scoring functions such as the proprietary Glide Score (Eq. 3) which is based on ChemScore [17] which describes the interactions between the atoms of the protein and ligand through a parameterized expression:



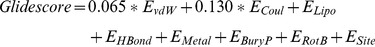
(Eq. 3)


Here, each term describes different types of interactions that are possible between a protein and a ligand. The terms are a van der Waals (vdW) energy or contact term, a Coulomb (Coul) interaction energy describing electrostatic interactions, the *Lipo* term that accounts for lipophilic interactions, the *HBond* term that governs hydrogen bond interactions, the Metal term that governs ligand-metal interactions, the *BuryP* term that penalizes buried polar groups, the *RotB* term that gives a penalty for freezing rotatable bonds and finally Site term that takes care of polar interactions in the active site, respectively.

The great advantage of scoring functions is the computational efficiency in terms of predicting correct binding poses as well as their ability to be relatively good at discriminating the binders from non-binders. However, the ranking of binders is predicted rather poorly using most of the scoring functions [10]. In the present study, we use a refined Glide Score where we replace the original metal term with a chelation energy term based on QM calculations.

### Preparation of Protein-Ligand Complexes

The ligands used in this study were obtained from our previous study [18] (the compounds were retrieved from the literature or the PubChem database). Both set of compounds (literature or PubChem) were converted to 3D structures using OMEGA (a conformation generating tool)[19]. By default OMEGA reports multiple conformers for all compounds using the MMFF94S force field, however, in the present study only a single i.e., the lowest energy conformer, was used. Subsequently, all compounds were preprocessed using the LigPrep module of the Schrödinger package [20] (energy minimization and determination of protonation states using the Epik tool).

**Figure 1:**
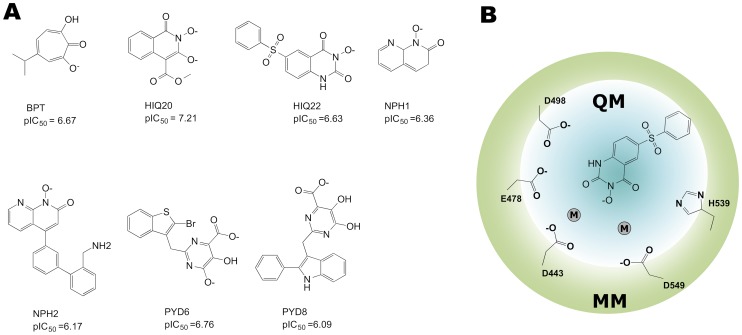
2D chemical structures of RNH inhibitors and QM/MM components. A. Compounds used in this study; B. The components of the QM and MM region for geometry optimization are shown.

A computational model of the HIV-1 RT associated RNase H domain was built from an X-ray crystal structure (resolution of 1.4 Å) from the Protein Data Bank (PDB ID: 3QIO) [21]. Missing residues were added using the Swiss-Model [22]. Subsequently, the atomic coordinates of the protein was imported into the Maestro module available in the Schrödinger package [23] and the protein was further optimized (e.g., adding hydrogen atoms, assigning correct bond orders, building di-sulfide bonds and replacing crystal bound Mn^2+^ ions with catalytically active Mg^2+^ ions) using the Protein Preparation Wizard [24]. The docking experiment was performed as described in our previous work [18]. Protein-ligand complexes of the compounds under investigation (Figure 1) were obtained from Glide docking with the SP scoring function [8] (Eq. 3). Glide provides three docking precision modes, namely, XP (extra precision), SP (Standard precision) and HTS (High-throughput screening) modes. Each mode are used in slightly different context, e.g., the HTS mode is used to screen a relatively large database (uses more restricted conformational sampling), the SP mode uses a softer scoring function that adapt at identifying ligands that have a reasonable propensity to bind in the receptor, and the XP mode uses a complete minimization, and scoring (and additional terms used over SP, e.g., solvation) from large ensembles of docking poses (requires more CPU time). Thus this mode is specially used for top-ranked compounds in the virtual screening protocol. In this study only the best ranked docking pose based on the SP mode was considered. Subsequent each complex was exported as a pdb file for the QM based calculations. Table 1 shows the different chelation calculation scenarios that were considered in this study. Here, scenario 1 is defined as MP2 (Møller–Plesset perturbation method) based chelation calculations with coordinates taken directly from the docking pose. In these calculations we include only the Mg^2+^ ions and the inhibitor. This makes the chelation energy calculation fast to evaluate. In scenario 2, a QM/MM geometry optimization is performed on the whole system. We include only the two Mg^2+^ ions and the inhibitor in the QM region and the rest of the protein is treated with MM. Subsequently, the optimized structure was used for an energy calculation using the Fragment Molecular Orbital (FMO) method (see below for details). In scenario 3, like in scenario 2, a QM/MM geometry optimization is performed. In addition to the two Mg^2+^ ions and the inhibitor, several key residues were added to the QM region (see below). The rest of the protein was treated with MM. Finally FMO single point energy calculations were performed on these geometry-optimized structures.

**Table 1:** Different chelation energy calculation scenarios used in this study.

Scenarios	Energy calculation method	Geometry optimization method	QM component in geometry optimization	Time
1^a^	MP2 or B3LYP	No	2 Mg^2+^, Inhibitor	∼10 min or 1–3 min
2^b^	FMO-MP2	B3LYP (QM); OPLS-2005 (MM)	2 Mg^2+^, Inhibitor	∼4 hrs.
3^b^	FMO-MP2	B3LYP (QM); OPLS-2005 (MM)	2 Mg^2+^, Inhibitor, D498, D443, E478, D549, H539	∼4 hrs.

a Time is for energy evaluation on 8 CPU cores.

b Time is for FMO-MP2 energy evaluation on 80 CPU cores.

### Simple chelation model based on MP2 calculations

In principle it is possible to calculate the binding free energies using *ab initio* methods, however, calculation of the free energy is difficult and even intractable for large systems. Thus, an approximation is often invoked where only the energy (not free energy) is calculated (Eq.4) and the temperature is assumed to be 0 K:



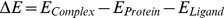
(Eq. 4)


The simplest possible model (scenario 1) to describe the binding of the ligand is to describe only the chelation process between the magnesium ions and the ligand in solution (as an approximation to a protein environment) yielding the following approximation to eq. 4



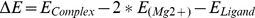
(Eq. 5)


For this scenario, only the atomic coordinates of the best-ranked docking poses and magnesium ions are extracted and used. All calculations in this scenario were carried out in Gaussian09 [25] using either MP2 or B3LYP [26], [27] and the 6-31G(d) basis set. [28]–[30] In this simple model where the entire protein is neglected, we have chosen to describe the chelation as taking place in solution with a dielectric constant of ε = 78.4 (water). This is modeled using the conductor like polarizable continuum model (C-PCM) [31]–[33]. In all calculations with C-PCM, we used a van der Waal radius of 1.5 Å for the Mg^2+^ ions. All radii were scaled by a factor 1.2 during the generation of the cavity. By changing the dielectric constant to 4 one can simulate better the similar process taking place in the protein, however, we found that this did not change the internal ranking of any of the tested compounds. Calculating the binding energy takes approximately 10 minutes per ligand using this simple chelation model. The simple approach outlined in scenario 1 above has some drawbacks although being a viable and feasible strategy for thousands of compounds: It lacks the direct inclusion of the protein which could have great influence on the ligand binding.

### QM/MM optimizations and all-protein calculations

To refine on scenario 1, we consider in scenario 2 geometry optimization of the protein-ligand complexes using the Qsite module (version 5.0) [34] of the Schrödinger suite. Here, the magnesium ions and inhibitors were considered in the QM region (optimized with B3LYP and the 6-31G(d) basis set). The rest of the protein was considered in the MM region (optimized using the OPLS-2005 force field) as shown in Figure 1B. In scenario 3, the inhibitors, the magnesium ions and five key residues (D443, E478, D498, D549 and H539) were considered in the QM region, as residues such as D443, E478, D498 and D549 are coordinated directly with the two Mg^2+^ ions which are necessary for substrate binding during the HIV reverse transcription process and furthermore the H539 residue has been shown to be essential for the ligand binding [18]. In both these scenarios, the geometry-optimized structures are used for the FMO calculations.

In the FMO method [35], the entire system is divided into several fragments and their energies are evaluated in the presence of all other fragments. This is known as the one-body FMO method (FMO1). Usually, a single fragment consists of a single protein residue. To further enhance the quality of the calculation and include important QM effects, all pairs of fragments are evaluated in the presence of the rest of the fragments. This is known as the two-body FMO method (FMO2). The total energy for an FMO2 calculation is given as (**6**)



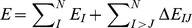
(Eq. 6)where E_I_ is the energy of a monomer in the electrostatic potential of all other monomers. ΔE_IJ_ is the interaction energy of fragment I and J evaluated as (**7**)



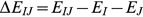
(Eq. 7)


To prepare the input files for FMO in GAMESS [36], we used FragIt [37], which is an automated tool to help with the fragmentation procedure. It is based on chemically driven simple rules about where to do the fragmentation. We used one residue per fragment throughout with the exception of D443, E478 and D549, which were combined into one single fragment with the two magnesium ions for a total fragment charge of +1.

This was done in order to increase the accuracy of the calculation. In FMO2, charge-transfer is described only between pairs of fragments, but the close proximity of the negatively charged residues and the positively charged magnesium ions will give rise to complex many-body interactions that FMO2 will fail to capture. By including these residues into one single fragment, these interactions are accounted for but with an added computational cost. We have chosen not to include D498 or H530 due to the computational scaling involved. Covalent bonds between fragments were treated using the Adaptive Frozen Orbital scheme [38]. All fragment calculations used MP2 [39], the 6-31G(d) basis set and C-PCM [40] to treat the effect of solvation with a dielectric constant of ε = 78.4.

## Results and Discussion

### Prediction of binding affinity by the simple metal reactivity model

Docking methods have been used successfully in two scenarios; one is accurate prediction of ligand binding poses, and another is to retrieve active compounds from a set of compounds. The latter is frequently being used in virtual screening processes.

Docking methods could also be used for ranking compounds, however, the correlation between scoring functions and experimental values for binding free energies is rather poor. One reason for this is the lack of protein flexibility in the majority of the docking simulations. In this investigation, we compare our simple metal reactivity model and FMO based chelation energy calculations with scoring functions e.g., the Glide Scores (SP and XP scores). We have randomly selected a set of seven RNase H inhibitors with known activity (IC_50_) for this study (Figure 1A). A summary of chelation energies calculated from the different scenarios is provided in Table 2.

**Table 2:** Summary of experimental and calculated energies of binding of HIV-1 RNase H inhibitors.

Compound name	pIC_50_	Glide Score (SP) (kcal/mol)	Chelation energy (kcal/mol)			Modified score^#^ (kcal/mol)
			Scenario 1	Scenario 2	Scenario 3	
Hiosquninone-20 (HIQ20)	7.21	−9.7	−164.8	−198.2	−184.4	−172.8
Pyrimidinolol-6 (PYD6)	6.76	−6.6	−124.5	−181.5	−155.4	−132.1
Beta-Thujaplicinol (BTP)	6.67	−9.9	−123.1	−170.9	−163.3	−130.9
Hiosquninone-22 (HIQ22)	6.63	−7.9	−116.7	−191.4	−157.5	−125.2
Naphthyridine-1 (NPH1)	6.36	−9.7	−105.2	−137.8	−114.9	−112.9
Naphthyridine-2 (NPH2)	6.17	−8.5	−104.2	−146.1	−121.8	−110.8
Pyrimidinol-8 (PYD8)	6.09	−7.8	−82.8	−137.6	−123.0	−88.6

**Note**: Scenario 1: Chelation energies obtained from MP2 calculations, Scenario 2: Chelation energies obtained from FMO-MP2 calculation with geometry optimization using QM/MM DFT-B3LYP, Scenario 3: Chelation energies obtained from FMO-MP2 calculation with geometry optimization using QM/MM DFT-B3LYP employing in extended QM region. ^#^Modified score is derived from the equation 8 (see text for detail).

**Figure 2:**
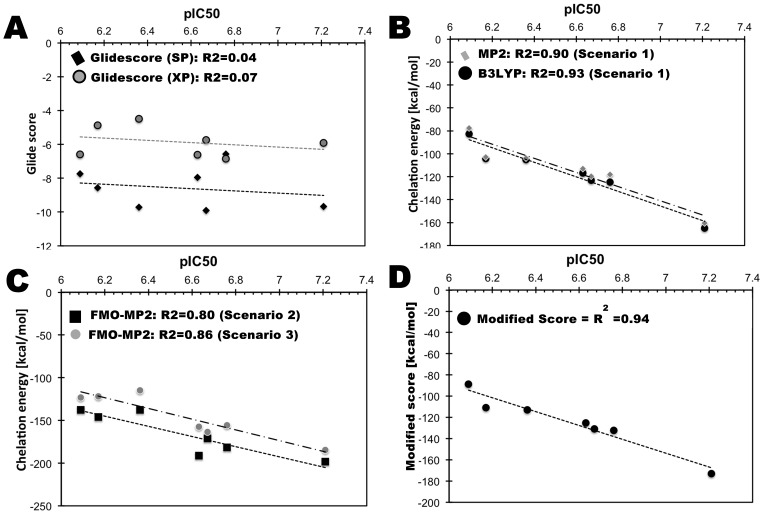
Correlation between observed pIC_50_ and predicted binding energy based on the Glide scores (A), scenario 1 (B), scenario 2 and 3 (C), and modified Chelation Score (D).

In Figure 2A we show the correlation between the Glide Score and the experimental activity. As expected there is no correlation between the docking score (both SP and XP scoring functions) and the experimental activity (R^2^ = 0.04 for SP and 0.079 for XP). However, when the QM based chelation is used (scenario 1, Figure 2B), one can clearly see that the experimental activity nicely correlates with the chelation energy with a coefficient of 0.90.

Before we discuss the binding energies obtained in scenario 2 using the FMO method, it is worthwhile to investigate the effect that the geometry optimization of the ligand and magnesium ions has on the protein-ligand complex. The position of the magnesium ions and ligands were compared with the initial stages of docking, and it is observed that upon QM/MM geometry optimization there is a slight change in the position of the magnesium ions (∼0.1−0.2 Å) and ligands compared to the initial positions. In some case, the position of the magnesium ions is closer to the ligands than in the initial stage. The distance between the magnesium ions is also slightly adjusted according to the position of the negatively charged ligands and the distance between the two magnesium ions are observed to be between 3.8 to 4.2 Å. The binding free energy values from scenario 2 yield relatively lower energies (−137 to −198 kcal/mol) than scenario 1 (−82 to −164 kcal/mol) i.e., a stronger binding is found in scenario 2. The observed correlation between scenario 2 predictions and experimental values is 0.80 (Figure 2C).

The chelation energy of compounds such as NPH1 (pIC_50_ = 6.36), NPH2 (pIC_50_ = 6.17) and PYD8 (pIC_50_ = 6.09) is slightly higher and could not be differentiated by scenario 2 compared to scenario 1. Although there is slight structural similarity between NHP1 and PYD8, the activities of these two compounds are quite different. On the other hand scenario 1 predicts these compounds slightly better in the sense that the compounds are ranked as they are ranked according to experiment. The chelation energy of these compounds (in scenario 1) are as follows, NPH1 (−105.19 kcal/mol) > NPH2 (−104.21 kcal/mol) > PYD8 (−82.75 kcal/mol).

In scenario 3, we considered the effect of protein residues in the QM/MM geometry optimizations; key residues D498, D443, E478, D549, H539, 2 Mg^2+^ and the ligand were geometry optimized with DFT-B3LYP (QM) and the rest of the protein residues were optimized with MM (OPLS-2005) (see Figure 1B). Subsequently, the optimized complexes for all ligands were analyzed. The position of the key residues of the optimized complex change significantly (average difference is ∼0.1–0.3 Å) from the initial positions. The optimized structures were used for FMO calculations. The correlation between the chelation energy from these optimized structures and experimentally observed values are found to be 0. 86 (Figure 2C) which is higher than what was found in scenario 2. It can be seen that when some of the protein side chains are included in the geometry optimization, the correlation between chelation energy and experimental activity is improved. However this calculation is computationally very expensive compared to scenario 2.

**Figure 3:**
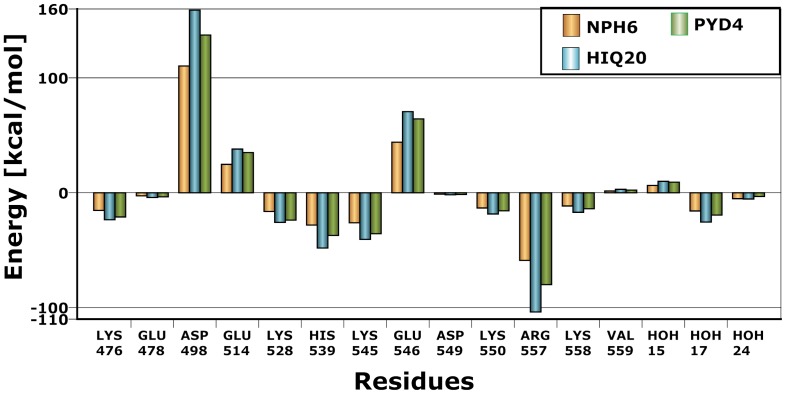
Residues contribution to Binding affinity. Residue energy contribution to the overall binding affinity from the FMO calculation (only significant numbers are shown) is shown for representative compounds. All values are provided in kcal/mol.

**Figure 4:**
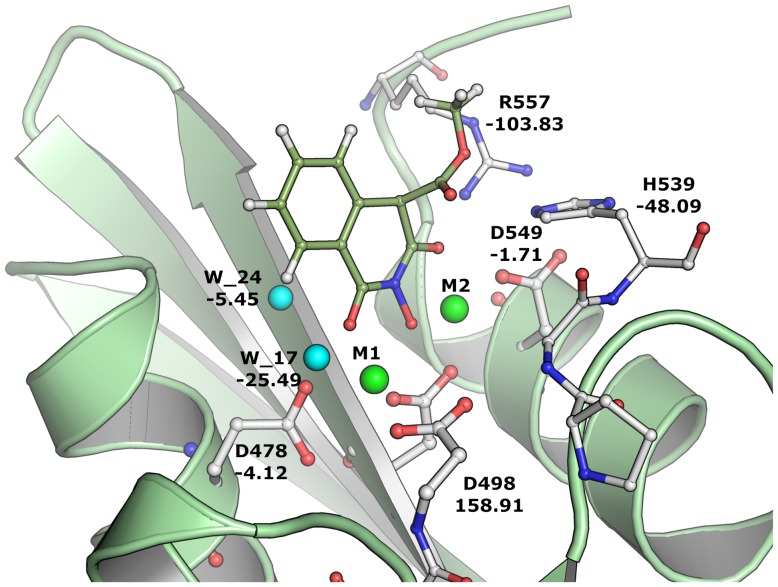
Binding mode of HIQ20 at RNH active site. Binding pose of HIQ20 at the RNase H catalytic site with important residues including water molecules (cyan sphere) and magnesium ions (green sphere). Energy contribution due to each residue is shown in kcal/mol based on the FMO calculation.

**Figure 5:**
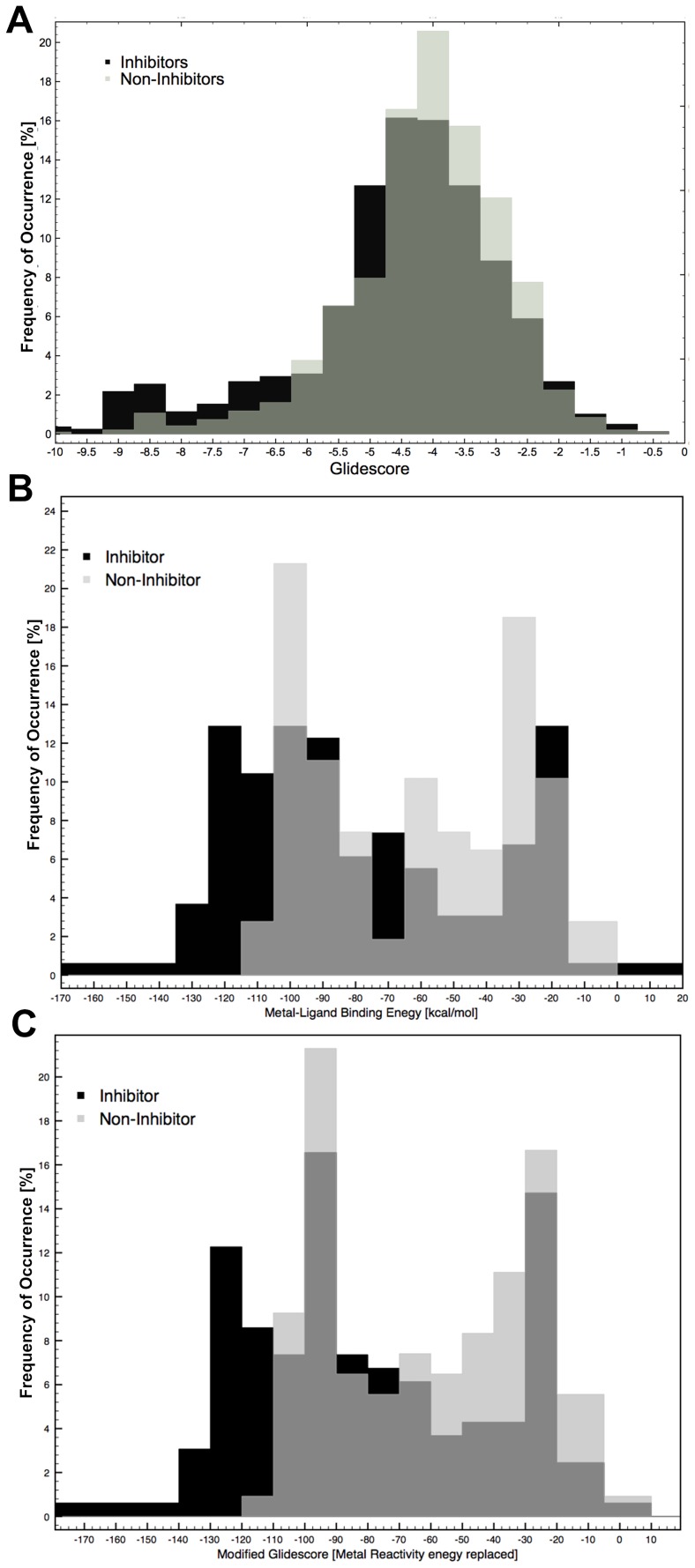
Distribution of binders and non-binders. A. Distribution of binders and non-binders based on the docking scores; B. Calculated chelation energy score; C. Modified Chelation Score.

It is very clear that these residues also play an important role in the positioning and reactivity of magnesium which favorably interacts with the inhibitors. These residues have previously been shown to play an essential role in the proper positioning of DNA:RNA duplex and anchor of magnesium ions (also some waters) in order to carry out the RNA removal from the hybrid structure. Although results based as the model in scenario 3 were improved significantly compared to scenario 2, scenario 1 is found to perform faster as the scenario 1 only takes around 10 minutes per ligand. However, scenario 2 and 3 takes the protein interactions explicitly into account meaning that at the end of the calculation, these methods produce energy contribution of each residue to the overall energy of the protein-ligand interaction energy. In principle, the interaction energy due to each residue could be useful in order to differentiate the activity based on favorable and unfavorable interactions of the residues (Figure 3). From a residue interaction energy analysis (Eq.7), it is found that residues D498 and E546 in all cases shown unfavorable energy contribution of +48 and +25 kcal/mol, respectively. This observation is interesting because it has previously been shown that any inhibitor that binds favorably with D498 leads to unwanted side effects due to its dual binding with human RNase H [41], [42]. Residues such as H539 (∼−20 kcal/mol), R557 (∼−45 kcal/mol), H_2_O17 and H_2_O24 possess very favorable interactions with all ligands. Previous studies also suggest that H539 plays an essential role in inhibitor binding [18]. In addition to H539 and R557, residue E514 also contributes very favorable to the overall binding energy. Residue R557 lies opposite to H539 and is involved in hydrogen bonding with ligands such as HIQ20 (IC_50_  = 0.061 µM), a strong binder in the dataset (Figure 4). As shown in our previous study [18] water molecules such as H_2_O17 and H_2_O24 also frequently participate in hydrogen bonding networks with a large number of inhibitors (13 out of 30 compounds). In Figure 5, we also observed favorable contribution (∼−10 kcal/mol) of these water molecules to the overall binding energy.

In addition to the MP2 based chelation energy calculations, we used also the DFT-B3LYP method for chelation energy calculation in scenario 1 (i.e., two Mg^2+^ ions and inhibitor from the docking pose), because this method is computationally less expensive than MP2 based energy calculations. It is clear from Figure 2B that the DFT-B3LYP based chelation energy nicely correlates with the experimental activity with a correlation of 0.93. Therefore, for the rest of the work described in this study, we use in scenario 1 DFT-B3LYP based chelation energy calculation.

Furthermore, we extended our analysis to refine the docking score (Figure 2D) by replacing the metal term in the Glide Score with the chelation term from scenario 1 to check if this helps in ranking the compounds. It is clear from Figure 2D that the correlation between experimental values and the refined Glide Score is significantly improved (from 0.098 to 0.93). The time scale for scenario 1 using DFT-B3LYP takes approximately 1–3 mins per ligand and one can easily replace this chelation term (*Chelation*) with the metal term of Glide Score for ranking the compounds, and subsequently it could be used in virtual screening for inhibition.
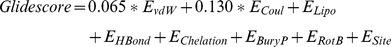



From the various scenarios studied, it is clearly observed that the QM based chelation (scenario 1–3) indeed improves the inhibitor ranking according to the experimental activities compared to using the standard Glide Score (Eq. 3). Thus, scenario 1 could be useful in either refining the Glide Score or as filter in the virtual screening of RNH inhibition, as this calculation is computationally efficient and relatively accurate. Moreover, this hypothesis could also be applied to the virtual screening of HIV-1 integrase inhibition as both enzymes share very similar inhibition mechanism e.g., metal-mediated catalysis. The strength of our proposed chelation score over the regular metal term in the Glide score is that the chelation score is a binding energy that is calculated from the energy difference between the bound and free confirmation of the ligand and magnesium ions. The metal term in the Glide score considers only the anionic or highly polar interactions between the ligand and metal in the complex. Thus, the chelation score captures all effects related to the chelation where as the metal term (in Glide score) only considers very specific interactions.

### Classification of binders and non-binders by scoring functions

In order to assess the applicability of the QM based chelation in a virtual screening context, we used scenario 1 for classification of binders and non-binders. Although most of the docking programs are not developed for binding affinity predictions, the scoring functions rank the tested compounds. Classifying if a compound is a binder (i.e. inhibitor or active) or a non-binder (i.e., non-inhibitor or inactive) to a given receptor is more difficult than screening for some binders in a set of non-binders (non-inhibitors) as studied in the enrichment analysis [43]. Here we try to classify binders and non-binders according to their docking score (e.g., Glide Score). As shown in our previous study [18] Glide Score with standard precision (SP) is shown to be good in discriminating known actives from inactive compounds. In addition, machine learning based classifiers (i.e., random forest) performed reasonably well in classifying inhibitors (71%) and non-inhibitors (73%) using 2D descriptors (Mathews correlation coefficient  = 0.44 and G-mean = 0.73) for the test set.

A dataset of 1656 compounds (PubChem source) were preprocessed and docked into the RNH binding site and subsequently docking poses were scored using the Glide Score (SP) [18]. The scoring values for the whole dataset ranges from −9.8 to 0.03 kcal/mol (the binder starts with −9.8 to −0.4 kcal/mol and non-binder starts with −9.67 to 0.03 kcal/mol). The scoring values of binder and non-binder were used to make a distribution curve in order to check how well the scoring function discriminates the binders and non-binders from each other (Figure 5A). As can be seen from the plot there is no clear discrimination based on the Glide Score, however, the significant amount of binders have good scoring values. From a virtual screening point of view reducing the number of false positive in the early screening phase is major challenge. Therefore, we tried to reduce the number of false-positives using the DFT-B3LYP based chelation energy calculation as it showed good correlation with the observed activity for the small test set (Figure 2B). The chelation energy of the binders and non-binders are used for making a distribution curve Figure 5B, and subsequently we checked if there is an improvement compared to scoring based classification (Figure 5A). Although there are still a considerable number of false positives, from the curve, one can clearly see that including the chelation energy indeed play an important role in highlighting the binders from the non-binders. The chelation energy for binders lies between −170 kcal/mol to +80 kcal/mol and for the non-binders between −125 kcal/mol and +80 kcal/mol. Looking at the number of compounds which lie on the favorable energy scale, a large number of the binders (>10%) have chelation energy between −170 to −80 kcal/mol and only a less number of non-binders (<5%) have energies between these ranges, and more importantly none of the non-binders have favorable energy <−125 kcal/mol.

In a successful virtual screening protocols, only less than 2% of the top ranked compounds are usually considered for pharmacological testing [44]–[46]. In this context, the chelation energy calculation could potentially be used to reduce a significant number of false positives in the HIV-1 RNH inhibition screening. However, it is emphasized here that chelation energy calculation works only when the compounds screened for bind at the active site and not allosteric mechanism based inhibitors. As we discussed, there is a reduction in the number of the false positive rate when involving the results based on the chelation energy calculation and we tried to refine the Glide Score by replacing the metal term with the calculated chelation energy. It is observed from Figure 5C that there is a slight improvement in the distribution of binders and non-binders as compared to Figure 5B. However, compared to the scoring based distribution, the refined scoring shows improved distribution and a significant amount of false positives is eliminated.

Possible reasons for the false predictions of some of the compounds in the Pubchem dataset may be that these compounds bind at the allosteric site because the screening assay used in the Pubchem bioassay compounds for this dataset was based on the inhibition of HIV-1 RNH catalysis, meaning that inhibition measurement includes both the allosteric and the active-site direct inhibition mechanism. Another possible reason might be that the inhibitors bind in a different conformation than the one that is predicted by the docking simulations. Furthermore, the protein conformation is considered to be rigid in the docking. It should also be emphasized that ligand protonation states play a crucial role in ranking of compounds.

Furthermore, the partial atomic charges of the ligands play an important role in the docking. Since, RNH contains two catalytically active magnesium ions in the binding site, if any compounds have a negative charge (e.g., carboxylate ions) then the docking algorithm predicts the ligands to bind strongly with the positive charged magnesium ions with a favorable docking score.

### Case study: Chelation energy calculation based virtual screening of RNase H inhibition

**Figure 6:**
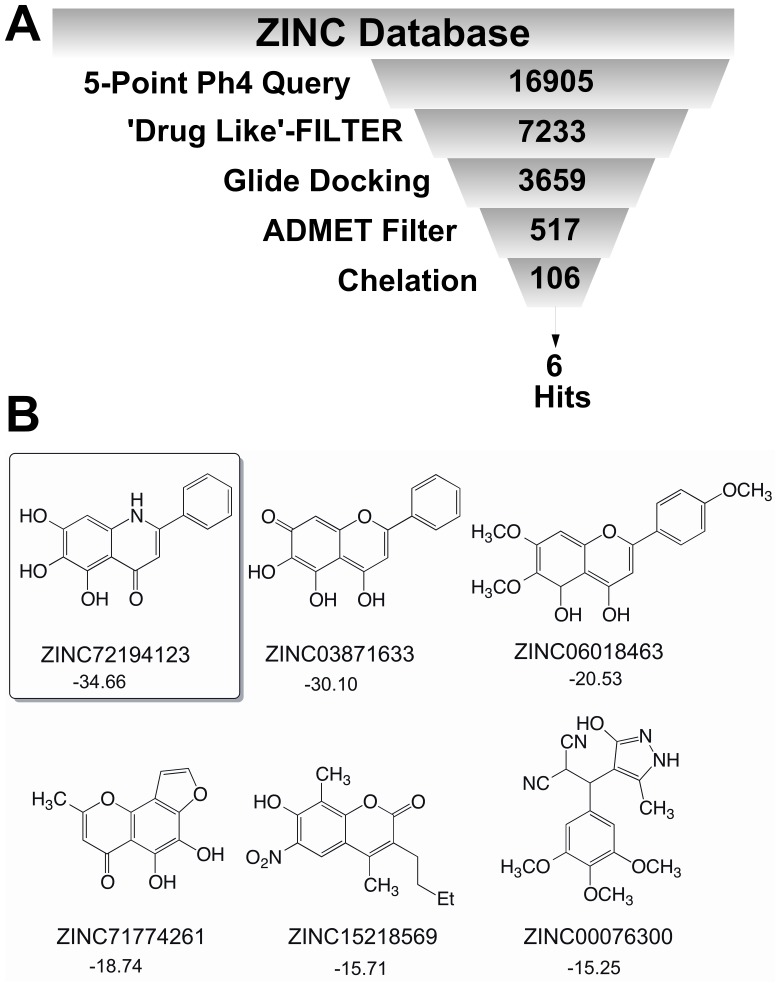
Models to identification. A. Virtual screening workflow; B. 2D representation of Hits obtained from the virtual screening process is shown with ZINC ID and chelation energy in kcal/mol.

**Figure 7:**
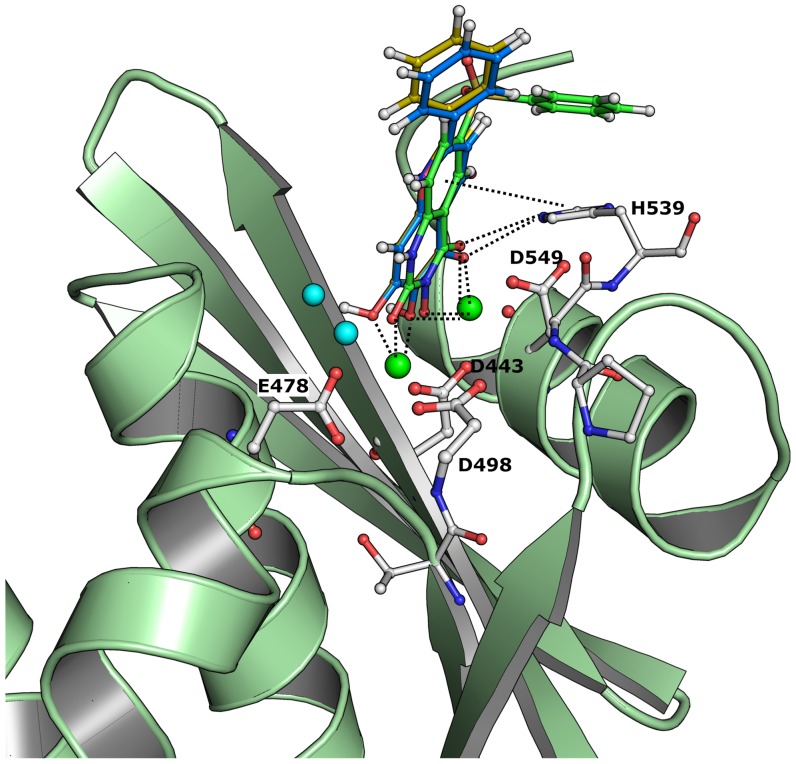
Comparison of Binding mode. Binding mode of ZINC72194123 (blue) and ZINC03871633 (yellow) is shown in ball and stick model with bound ligand (green). Important residues are highlighted, including magnesium ions (green sphere) and water molecules (cyan sphere).

In order to assess the chelation calculation as a useful approach for virtual screening, we further tested the chelation calculation (scenario 1) with the ZINC Pharmer as a compound source [47]. The overall workflow of the virtual screening is shown in Figure 6A. Based on the previous study [18], we defined a five-point pharmacophore query for compound filtration in the ZINC Pharmer web tool, which results in 16905 compounds. After ligand preprocessing steps such as the 3D conformation generation (using the OMEGA tool from the OpenEye suite)[19], prediction of protonation states (using the Epik tool from the Schrodinger suite) and drug-like filter (using the FILTER program from the OpenEye [48]) all the compounds were docked into the RNH active site using Glide SP. Out of 3659 docked compounds, only 517 compounds had high docking scores (less than −6.00 kcal/mol), which were then manually checked with ADMET (absorption, distribution, metabolism, excretion and toxicity) filtration (properties generated from the QuickProp tool from the Schrodinger suite)[49] which results in 107 compounds. This set of compounds was used for the chelation calculation (using scenario 1). Of the 107 compounds, only six compounds had favorable chelation energy (a chelation energy less than zero kcal/mol) (Figure 6B). Hits such as ZINC72194123 (common name *Baicalein*) and ZINC03871633 have favorable docking score (<−7.00 kcal/mol) and chelation energy (<0 kcal/mol). The binding mode of these compounds revealed that both compounds are strongly coordinated with magnesium ions and hydrogen bond/π with His539. The overall structural features of these hits are very similar to the known RNH inhibitors as the majority of inhibitors possess a three-oxygen pharmcophore that strongly binds with magnesium ions, e.g. pyrimidinone, diketo, tropolone, N-hydroxyimide, diones [16]. The binding mode of the top hits is shown in Figure 7. In addition, from the literature we found that, Baicalein (a flavonoid compound) originally was isolated from Chinese herbal medicine (*Scutellaria baicalensis Georgi*) and potentially inhibits HIV-1 replication through various inhibition mechanisms e.g., integrase (possess similar binding site as RNH)[50], reverse transcriptase [51] and HIV-1 env [52] inhibitors. More importantly, Baicalein has not yet been reported as a RNH inhibitor, however, it has been reported as a nonspecific reverse transcriptase inhibitor. Therefore, we believe that Baicalein reverse transcriptase inhibition activity may be due to the effect of RNH inhibition and not polymerase inhibition, as this compound strongly binds with magnesium ions as it is binds with intergase enzyme as shown before [50].

From this virtual screening, it is noted that the simple chelation calculation (scenario 1) in addition to the docking experiments potentially eliminates the false positives and significantly improves the virtual screening success rate especially for HIV-1 reverse transcriptase associated RNase H inhibition.

## Conclusions

In the present investigation we have developed a high-throughput filter based on chelation energy calculations using a QM based method. The compounds are ranked based on chelation energy calculations, which outperform the ranking based on the use of a conventional scoring function. Overall, these results show that the simple chelation model (scenario 1) is a very promising method for predicting the binding affinity of a set of known ligands, although it needs to be validated on a larger set of known inhibitors. A drawback of this model is that it can only be used for HIV-1 RNH active site directed binders, (e.g., not for allosteric inhibitors) and rely on the accuracy of the docking pose. We are currently performing work to see if this simple/optimized chelation models could be further improved with large dataset of RNase H active site binders and also try applying this protocol to other metal containing proteins.
